# Analysis of zebrafish (*Danio rerio*) behavior in response to bacterial infection using a self-organizing map

**DOI:** 10.1186/s12917-015-0579-2

**Published:** 2015-10-23

**Authors:** Sang-Bin Lee, Yunjeong Choe, Tae-Soo Chon, Ho Young Kang

**Affiliations:** Department of Biological Sciences, Pusan National University, Busan, 609-735 Republic of Korea; Department of Microbiology, Pusan National University, Busandaehak-ro, 63beon-gill, Geumjeong-gu, Busan, 609-735 Republic of Korea; Ecology & Future Research Association (EnFRA), 21 Dusil-ro, 45 beon-gil, Geumjeong-gu, Busan, 609-802 Republic of Korea

**Keywords:** Computational behavior, Diagnosis, Fish disease, Fish pathogen, *Edwardsiella tarda*, Movement patterns

## Abstract

**Background:**

Animal behavioral responses have been recently established as a suitable tool for detecting contaminants in the environment for risk assessment *in situ*. In this study, we observed movement behavior of zebrafish (*Danio rerio*) before and after infection with *Edwardsiella tarda* CK41 for 3 days until death.

**Methods:**

Infection status of zebrafish was confirmed through PCR and colonization assay as time progressed and lesion development in the tails of zebrafish was also examined. Movement behaviors in response to bacterial infection were patterned by self-organizing map (SOM) based on movement parameters, including speed (*mm/s*), acceleration (*mm/s*^*2*^), stop duration (*t*), stop number (*n*), locomotory rate (*mm/s*), turning rate (*rad/s*), and meander (*rad/mm*).

**Results:**

According to SOM result, clusters were identified firstly according to time and secondly according to infection. Two movement patterns were observed in the early period of infection: one group with minimum turning rate and meander (i.e., stiff movement) and the other group with maximum strop number. Late infection was characterized by long stop duration.

**Conclusion:**

SOM was suitable for extracting complex behavioral data and thus can serve as a referencing system for diagnosing disease development in order to reveal the mechanism of the infection process.

**Electronic supplementary material:**

The online version of this article (doi:10.1186/s12917-015-0579-2) contains supplementary material, which is available to authorized users.

## Background

Along with development of interfacing techniques [1, 2] and computational approaches, response behaviors of indicator species have been used for monitoring stressful conditions since the early 1990s [3–8]. In accordance with computer technology development, image processing and recording systems have also been developed concurrently for tracking [9, 10]. Continuous movement data, however, are difficult to analyze due to the non-linearity of behavioral data, high levels of noise, and huge amounts of data. Although there have been numerous reports conducted on behavior monitoring, response behaviors after pathogenic infection have been seldom examined [1, 2, 11, 12] Nonetheless, behavior monitoring has recently garnered attention since concurrent understanding of host behaviors and pathogenic development would help elucidate useful host-pathogen causality relationships and integrative diagnosing systems.

Many fish species, including fishery products, are infected by pathogenic microbes, resulting in serious losses to fish productivity worldwide [13]. Epidemiological approaches can be conducted in order to analyze the mechanism of infection through an ecological viewpoint. However, epidemiology mainly concerns information at the population level [14]. Pathophysiological alteration can be assumed from the behavior of infected fish, including important behaviors such as swimming performance and movement patterns [15]. Behaviors could provide information on the totality of changes at the individual level since behavior integrates all inner processes of physiological and genetic networks. Consequently, behavioral responses would add an additional dimension in diagnosing disease development in addition to physiological (e.g., molecular toxicology) [16] and ecological (e.g., epidemics) [17] approaches. Moreover, behavioral monitoring could fill in the gap between micro-scale (i.e., genetic level) and macro-scale (i.e., population/community level) monitoring considering the efficiency in continuously tracing diseased organisms.

Fish exposed to pathogen infection exhibit characteristic behavioral patterns corresponding to extensive toxicological symptoms, including hemorrhages, neurological instability, and endocrine abnormality [18]. Abnormal behaviors such as anorexia, loss of equilibrium, erratic swimming, disorganized swimming, and slow motion would be thus expected [17, 19]. However, very little is known about fish behavior in response to infection with bacterial pathogen, especially behavioral pattern changes during the course of infection until death [20]. Although a few accounts of fish behavior have been reported in response to a viral agent such as rhabdovirus, koi herpes virus, nodavirus, and iridovirus [19, 21, 22], bacterial infection in fish has received relatively little attention. Abnormal swimming behavior was recognized in salmon species infected with the virulent bacterium, *Piscirickettsia salmonis*, manifesting weaker signs such as swimming near the surface or the edges of cage [23]. However, this response behavior was observed within a fixed time after bacterial infection.

*Edwardsiella tarda* is an enteric gram negative bacterium that causes an opportunistic infection in fish known as edwardsiellosis [24]. This disease is related to water conditions and stress [25], and it causes high fatality and tremendous economic losses in farming fishes such as flounder [26], eel [27], salmon [28], catfish [29] and others [30]. *E. tarda* accesses fish through the mucosal route, including the skin, gills, and intestine [31]. Several fish species can acquire *E. tarda* through immersion upon dermal abrasion [32, 33]. *E. tarda* as an intracellular pathogen can also invade internal organs such as the spleen, liver, and kidney [34], colonize epithelial cells and macrophages, and proliferate in cells for systemic infection [35–37]. These features are characteristics of *E. tarda* pathogenesis.

Zebrafish has become a popular experimental tool for studying the ethology, immunology, oncology, and toxicology of human disease [38]. In particular, zebrafish is a useful model for understanding aquatic pathogens such as *Staphylococcus aureus* [39], *Pseudomonas aeruginosa* [40], *Mycobacterium marinum* [41], *Streptococcus iniae* [42], and *Edwardsiella tarda* [33]. Further, zebrafish has been utilized for colonization of hosts [43] at different stages, including embryo and adult stages [44]. Various inoculation methods such as bath immersion have been successfully applied to zebrafish as a natural infection route [42, 45, 46]. Consequently, the advantages of zebrafish make the species an ideal infectious disease model for study of aquatic pathogen infection.

In this study, we recorded continuous movement of fish until death after infection with a fish pathogen, and movement data were analyzed by an information technique based on an artificial neural network (i.e., self-organizing map). We found that behavior patterns could be identified during the course of disease development and could be objectively characterized by parameters. Behavioral monitoring would be feasible in establishing a referencing system to diagnose disease development.

## Methods

### Model organisms and infection

#### Bacterial strains and media

*E. tarda* CK41 was used as the pathogenic bacteria in this study (Table 1). The bacterium was cultured in Tryptic Soy Broth (TSB) (Difco, USA) medium at 28 °C or was grown on TSB agar. Kanamycin antibiotic was added to the culture media at a concentration of 20 μg/ml. Bacterial cells were cultured for 8 h in TSB broth up to OD_600 nm_ 0.9-1.0. Bacterial strain *Aeromonas hydrophila* was used as an experimental control in PCR analysis and it was grown at 30 °C in TSB medium.

**Table 1:** Bacterial strains used in this study and calculated LD_50_ values

Strain	Characteristic	LD_50_ value (CFU/200 ml)	Reference
*E. tarda* CK41	pathogenic (isolates from diseased flounder)	1.14 × 10^10^	NFRDI^a^
*A. hydrophila*	pathogenic (isolates from tin of milk with a fishery odor)	─	KCTC2358^b^

^a^The National Fisheries Research and Development Institute (NFRDI) in the Republic of Korea

^b^The Korean Collection for Type Culture (KCTC) in the Republic of Korea

#### Experimental animal

Healthy zebrafish, *Danio rerio*, were purchased from a local dealer in Busan, Republic of Korea. Zebrafish were reared for 3 months before observation, and all fish were acclimated for 2 weeks under laboratory conditions. The light system was turned on at 7:00 h and off at 21:00 h (14/10 h light/dark cycle). Stock fish were maintained in aerated tanks supplied with static susceptible water (25 ± 1 °C). Fish were fed two times a day with commercial zebrafish feed, BIOGRAN (Prodac, Italy). The dechlorinated water was changed every other day. Healthy male zebrafish (2 ± 0.5 cm and 0.4 ± 0.05 g) were randomly selected from a stock population and transferred to acryl aquarium (15 cm × 15 cm × 10 cm, 5 cm (height of water)) for observation. Zebrafish were immersed into tricaine (ethyl 3-aminobenzoate methanesulfonate salt) (Sigma, USA) 0.01 % solution to reduce the pain and stress on the zebrafish. Clinical signs of infected zebrafish were examined by a Motic stereomicroscope SMZ168 (Motic, Hongkong) at 1.5X magnification.

#### Ethical statement

This study was conducted in strict guideline for the care and use of laboratory animals. Experimental treatment protocols were approved by the Pusan National University Institutional Animal Care and Use Committee (PNU-IACUC) (Approval Number: PNU-2015-0785) with respect to ethical issues and scientific care. Under the PNU-IACUC approved protocol, humane endpoint criteria were followed during this study and zebrafish were sacrificed with euthanasia. Bacterial inoculation was performed under tricaine anesthesia to minimize suffering and stress.

#### Experimental infection

Cultured *E. tarda* cells were harvested by centrifugation (5,000 × *g*, 10 min, 25 °C) and resuspended in phosphate-buffered saline (PBS, pH 7.4). Bacterial concentrations were adjusted by using a spectrophotometer. Viable bacterial cells were counted by culture on agar plates after serial dilution. Dosage levels used in the experiment were determined based on the results of LD_50_ measurement (Table 1). Zebrafish were individually inoculated by immersion (IM) route with diluted bacterial cells in 200 ml of PBS. IM challenge was carried out as described by Petrie-hanson *et al*. [47] with slight modifications; slight scar was created using an ultra-fine II syringe (BD, USA) before inoculation, followed by IM in an inoculation bath for 30 min with aeration. As natural infection route in a fish farm, this modified method was involved in this study. PBS bath was separately used for the control group. All fish were monitored for at least 5 days post-challenge.

### Colonization assay and PCR analysis for detection of bacteria

#### Enumeration of *E. tarda* in zebrafish

Zebrafish were killed by over-dose treatment of tricaine. Colonization and persistence of *E. tarda* CK41 were separately determined in the intestine, spleen, and kidney at 0.1, 1, and 2 days post-challenge, respectively. Organs and tissues from each zebrafish were dissected and homogenized using a homogenizer (IKA-Werke Gmbh & Co., Germany) for 60 s at 3,000 rpm, followed by PBS (1 ml) addition. Samples were serially diluted and dropped on TSB agar plates supplemented with the appropriate antibiotic to examine the number of viable *E. tarda* cells.

#### PCR amplification

PCR assay was used for detecting *E. tarda* infection from colonization isolates. The target region and oligonucleotide primer set used for PCR detection are indicated in Table 2. PCR was performed in 20 μl reaction mixtures containing sample template from each time point, 0.05 μM concentration of each primer (Bioneer, USA), and 2 × EF-Taq PCR mix (SolGent Co. Ltd., Republic of Korea). Amplifications were carried out in a Swift Max Pro thermocycler (Esco Healthcare, Singapore) with the following procedure: initial denaturation step at 94 °C for 5 min; 25 serial cycles of denaturation step at 94 °C for 30 s, annealing at 50 °C for 30 s, and extension at 72 °C for 3 min; and a final extension step at 72 °C for 7 min. A specific primer set for *A. hydrophila* 16 s rRNA sequence was included in this PCR to detect *A. hydrophila* used as a positive experimental control. The PCR products were analyzed by 0.8 % agarose gel electrophoresis in 1 % Tris-acetate-EDTA (TAE) buffer and visualized by staining with a SafeView™classic (Applied Biological Materials Inc., Canada). The gels were certificated using an ImageQuant LAS 500 (GE Healthcare, USA).

**Table 2:** Primer pairs for target genes used in this study

Bacteria	Target gene	Product size (bp)	Nucleotide sequence (5’ to 3’)	Reference
*E. tarda*	*gyrB1*	415	F: GCATGGAGACCTTCAGCAAT	[70]
			R: GCGGAGATTTTGCTCTTCTT	
*A. hydrophila*	*16S rDNA*	685	F: GAAAGGTTGATGCCTAATACG	[71]
			R: CGTGCTGGCAACAAAGGA	

### Computational behavior

#### Recording, tracking, and parameter calculation

The behavior observation system used in this study consisted of a transparent aquarium (15 cm × 15 cm × 10 cm), camcorder (SONY CX-700, Japan), image processing system (Virtual Dub software, http://www.virtualdub.org/), computer (Intel® Core ™ 2 Duo CPU E4500@ 2.20GHz), and white LED light. During observation, the system was covered with black fabric (cotton) to ensure stable recording without external noise. The light was placed below the aquarium [2]. Between the light source and aquarium semitransparent acryl (30 cm x 30 cm x 2 mm (thickness)) provide even distribution of light intensity to arena. Control and infection groups were recorded for 3 days each. For the control individuals, PBS was administered to fish instead of *E. tarda*. Infection groups were recorded until death of fish, if death occurred. Twenty-nine zebrafish were infected with *E. tarda* cells and their behaviors examined. Twenty two individuals were tested for the control.

After 10 min of acclimation in the arena, movement of zebrafish was recorded for 1 h using a digital camcorder every day from 10:10 AM to 11:10 AM. Multi-tracker was used for interfacing and recording positions of tested individuals [48, 49]. Although longer duration of recording can help to gain more information, it reduces the efficiency to extract valuable data. Considering the amount of data and the efficiency of analysis, a one-hour recording time was sufficient to obtain necessary data in the multiple preliminary studies using various recording times. Based on the results described in preliminary studies [2], a time segment of 0.25 s was selected for recording. In this study, our goal was to observe overall movement of zebrafish after infection, and 0.25 s segment was sufficiently short to observe displacement of each organism’s location [1, 12]. Once position data were collected, parameters were calculated in the MATLAB environment (The Mathworks, R2009). According to previous research [2] and preliminary studies, we calculated seven parameters (i.e., speed (*mm/s*), acceleration (*mm/s*^*2*^), stop duration (*t*), stop number (*n*), locomotory rate (*mm/s*), turning rate (*rad/s*), and meander (*rad/mm*)) (Table 3).

**Table 3:** Definition of each parameter

Parameter	Unit	Description
Speed	*mm/s*	Speed of zebrafish each 0.25 s
Acceleration	*mm/s* ^*2*^	Acceleration of zebrafish each 0.25 s
Stop duration	*t*	Stop time before zebrafish move duration between two consequent stops
Stop number	*n*	The number of zebrafish stop for a period
Locomotory rate	*mm/s*	Movement distance divided by the total movement time
Turning rate	*rad/s*	Total angle change per movement time
Meander	*rad/mm*	Total angle change per movement distance between two segments

#### Self-organizing map and statistical analysis

Complex movement behaviors were patterned using a self-organizing map (SOM) [50–52]. SOM performs unsupervised learning [53] of data without prior knowledge by compressing multi-dimensional data (e.g., movement parameters) into a few dimensions (conveniently 2). In the SOM, a linear array of M^2^ artificial neurons consisted of two layers (input and output). Initially, weight was randomly distributed in small values. Each neuron in the network calculates the summed distance between weights and distance d_j_(*t*) at output node *j*:

1 $$ {d}_j(t)={\displaystyle \sum_{i=0}^{s-1}{\left({x}_i-{w}_{ij}(t)\right)}^2} $$

where *x*_*i*_ is the value of the *i*-th parameter, *w*_*ij*_(*t*) is the weight between the *i*-th parameter and *j*-th node on the SOM. The neuron responding maximally to a given input vector is selected as the wining neuron, the weight vector of which has the shortest distance to the input vector. The winning neuron and possibly its neighboring neurons are allowed to learn by changing the weights in a manner that further reduces the distance between the weight and input vector. Input data were normalized by min-max normalization and consisted of sample units with variables (i.e., parameters).

2 $$ {w}_{ij}\left(t+1\right)={w}_{ij}(t)+\eta (t)\left({x}_i-{w}_{ij}(t)\right){Z}_j $$

where *η*(*t*) is learning rate and *t* is iteration time. The radius defining the neighborhood is usually given as a larger value early in the training process but is gradually reduced as convergence is reached.

Seven parameters stated above were used for training with individual sample units. In this study, SOM training was performed in two ways. Initially, clustering of sample units was conducted based on variables as presented in “Q” mode. Data matrix consisted of movement segments (158 (sample units) × 8 (variables)). Subsequently, the data matrix was transposed and clustering of variables performed in “R” mode (See details in Legendre and Legendre [54]). A map size of 9 × 7 in Q mode was based on the optimal number of map units (5$\sqrt{a}$; *a* is the number of training samples) [55]. Similarly, 4 × 4 map size was used in R mode. The SOM process was conducted using the SOM Toolbox [56] developed by the Laboratory of Information and Computer Science at Helsinki University of Technology (http://www.cis.hut.fi/projects/somtoolbox/).

## Results

### *E. tarda* infection in zebrafish

**Fig. 1:**
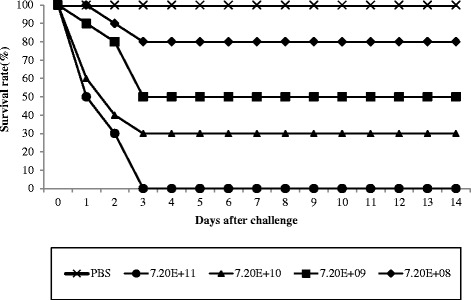
Survival rates of zebrafish inoculated with different dosage levels of *Edwardsiella tarda* CK41 as time progressed. *E. tarda* CK41 cells from fresh culture were administrated to four groups of zebrafish (n = 20 in each group) at doses of 7.2 × 10^8^ CFU/200 ml (◆), 7.2 × 10^9^ CFU/200 ml (■), 7.2 × 10^10^ CFU/200 ml (▲), and 7.2 × 10^11^ CFU/200 ml (●) by immersion route. Control zebrafish were inoculated with only PBS 200 ml (×). Mortality observations were recorded daily for an additional 14 days post-infection. Dose was determined by viable counting of bacterial cells after administration

**Fig. 2:**
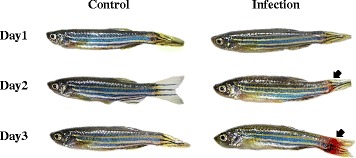
Gross pathology of infected zebrafish with *Edwardsiella tarda* CK41. Zebrafish immersed with *E. tarda* 1.0 × 10^11^ CFU / 200 ml for 30 min and subsequently examined at 3 days post-challenge for signs of infection and clinical disease. Arrow indicates hernia and dot hemorrhage from surrounding route of infection. Control zebrafish were inoculated with only 200 ml of PBS

The survival rates of IM-inoculated zebrafish were measured in order to evaluate the suitability of zebrafish for monitoring behavior in response to *E. tarda* infection. Initially, LD_50_ was calculated for a set of infection dosage levels. As seen in Fig. 1, dose-dependent survival rates were observed according to the dosage level from 2–3 days post-challenge. All zebrafish administered PBS survived during the 14 day post-challenge period (Fig. 1). Infection doses of 7.2 × 10^8^ CFU/200 ml, 7.2 × 10^9^ CFU/200 ml, and 7.2 × 10^10^ CFU/200 ml after 3 days post-challenge resulted in 80, 50, and 30 % survival, respectively. Infected zebrafish inoculated at the tail exhibited external signs of illness, including herniation and dot hemorrhaging, as time progressed (Fig. 2). All zebrafish died when inoculated with the maximal dose (7.2 × 10^11^ CFU/200 ml). The LD_50_ upon IM-inoculation was calculated as 1.14 × 10^10^ CFU/200 ml. Since we intended to examine fish behavior during the course of infection until death, we selected an infection dose of 1 × 10^11^ CFU/200 ml (10-fold higher than LD_50_) to guarantee observation from disease occurrence to initiation of death.

### Colonization of *E. tarda* in zebrafish

**Fig. 3:**
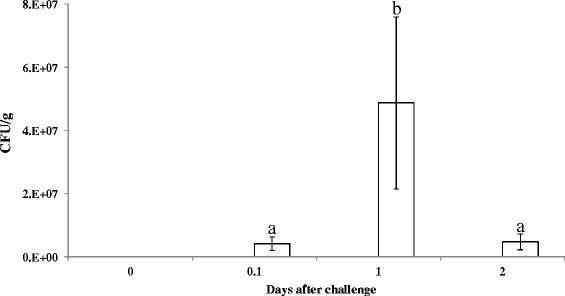
Colonization of zebrafish infected with *Edwardsiella tarda* CK41. Axenic zebrafish were immersed with *E. tarda* 1.0 × 10^11^ CFU / 200 ml (□), and PBS 200 ml (■) respectively. These zebrafish were dissected and their organs homogenized for viable counting. Bacteria were counted by plating onto TSB agar supplemented with the appropriate antibiotic. Standard deviations are indicated with vertical bars on the histogram (*n* = 10). Different alphabets listed above the bars indicate significant difference based on Tukey’s test (*p* < 0.05)

**Fig. 4:**
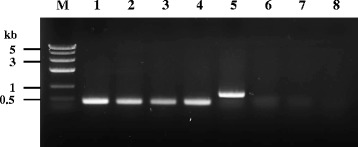
PCR amplification as time progressed after inoculation with colonization isolates to confirm *Edwardsiella tarda* infection. Specific primer pairs were used for detecting *E. tarda gyrB1* (415 bp) in Lanes 1–4 and *Aeromonas hydrophila* *16S rRNA *(685 bp) in Lane 5–8. Lane M, DNA ladder; Lane 1, *E. tarda* CK41; Lane 2, sample from day 0.1; Lane 3, sample from day 1; Lane 4, sample from day 2, Lane 5, *A. hydrophila*; Lane 6, sample from day 0.1; Lane 7, sample from day 1; Lane 8, sample from day 2

Bacterial colonization within the host is critical for confirming *E. tarda* infection. To assess the ability of *E. tarda* to persist within zebrafish, we measured the number of *E. tarda* CK41 cells in organs of zebrafish after IM-inoculation with an average dose (1.0 × 10^11^ CFU /200 ml). *E. tarda* CK41 cells were detected as early as 0.1 days post-challenge and increased until 1 day post-challenge*.* Bacterial infection subsequently decreased upon 2 days post-challenge (Fig. 3). This change in CFU/g number during the time course of infection suggests that *E. tarda* CK41 cells triggered a pathological response. To verify colonization of *E. tarda*, an *E. tarda*-specific gene was amplified by PCR using a set of specific primers (Fig. 4). The results of colonization assay suggest that *E. tarda* survived and multiplied in zebrafish organs at least 1 day post-challenge. These results are in accordance with the prior observations in the virulence assay (Fig. 3).

### Outline of movement behavior

**Fig. 5:**
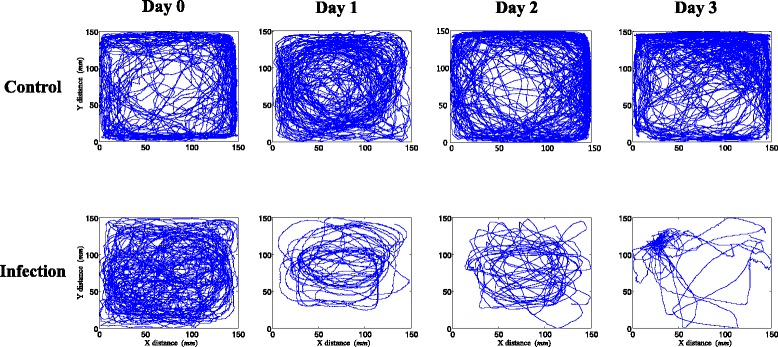
Movement tracks (30 min) of zebrafish after inoculation with *E. tarda* (Top panel; control, and bottom panel; infection)

A number of individuals died during the course of infection (3 individuals on 1 day, 17 individuals on 2 day, and 6 individuals on 3 day). The remaining living individuals were monitored to analyze movement behavior after infection. Figure 5 shows examples of movement tracks (30 min) for the control and infected individuals as time progressed. Movement patterns were different between the infected individuals and the control group. The control fish group moved broadly around the observation arena throughout the observation period (top panel, Fig. 5). The activity of infected zebrafish substantially decreased even after 1 day post-infection (bottom panel, Fig. 5). It is noteworthy that infected fish tended to make slow circling movements inside the arena within a narrow range (i.e., less-visit to boundary) along with intermittent stops in the late period. At 3 days post-challenge and close to death, the infected fish mostly stayed in one area for a long time and rarely moved outside this area, intermittently moving away from the original area to return back to the same area again (bottom right panel, Fig. 5).

### Patterning by SOM

**Fig. 6:**
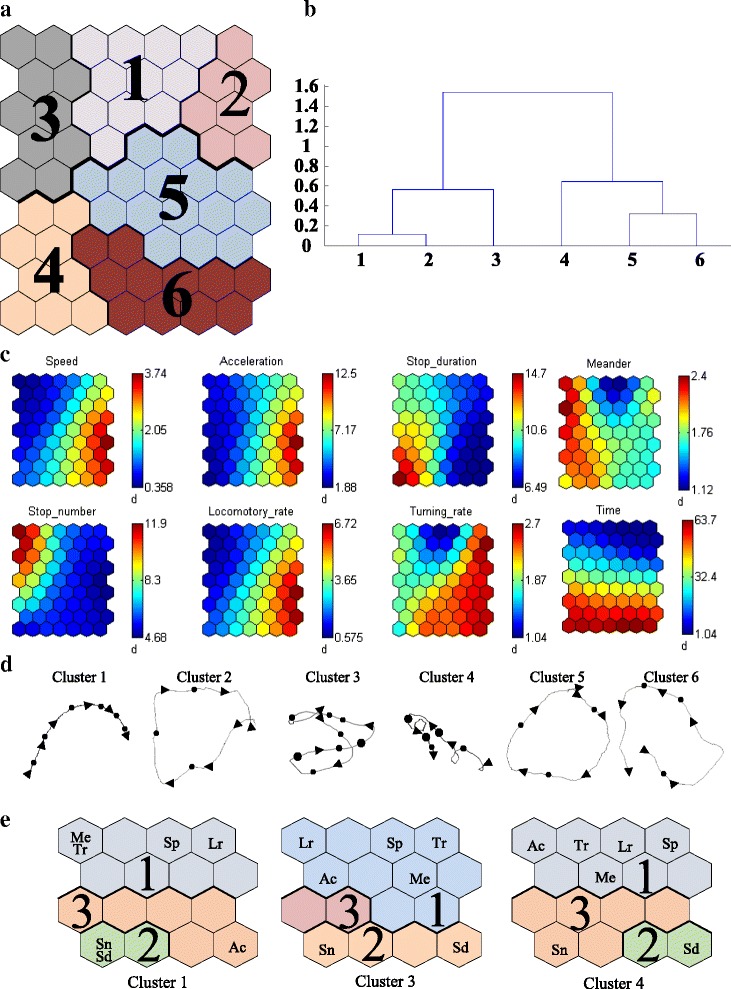
Movement clustering of zebrafish by SOM based on parameters after inoculation with *Edwardsiella tarda*. **a** Cluster, **b** dendrogram according to the Ward linkage method, **c** profile of parameters on the trained SOM (i.e., speed (*mm/s*), acceleration (*mm/s* ^*2*^), stop duration (*t*), stop number (*n*), locomotory rate (*mm/s*), turning rate (*rad/s*), and meander (rad/mm), **d** movement tracks for 10 s (black circles indicate stop duration according to size, arrows stand for velocity), and **e** association of parameters according to R mode (Me : meander, Tr : turning rate, Sp : speed, Lr : locomotory rate, Sn : stop number, Sd : stop duration, and Ac : acceleration)

According to the Ward linkage method, six clusters were observed on the SOM (Fig. 6a and b). Based on the parameter profiles exhibited on the component planes (Fig. 6c), the vertical gradient was presented mainly in accordance with “time”, suggesting that “time” is a major contributor for determining data variation in movement patterns: upper group of clusters 1, 2, and 3 and lower group of clusters 4, 5, and 6 (Fig. 6b). Movement parameters were further specifically variable along the horizontal gradient; speed, acceleration, and locomotory rate were high at the right position, whereas stop duration and stop number were high at the left position (Fig. 6c). Low levels of meander and turning rate were also observed locally within the upper group (Fig. 6c). Considering the movement patterns, clusters corresponding to the left position on the SOM in either the upper or lower group were associated with infected fish (e.g., slow speed, high meander, and high stop number/duration).

Subdivision was further observed within the vertical group (Fig. 6a). The upper group was divided into clusters 1, 3 and 2. Considering movement profiles (Fig. 6c), clusters 1 and 3 in the left matched with infection, whereas cluster 2 in the right was associated with control as stated above. In cluster 2, turning rate, locomotory rate, speed, and acceleration were high while stop duration and number were in the minimal range (Fig. 6c), presenting a healthy state. However, clusters 1 and 3 represented infection based on low active rates with reversed parameters values as compared to cluster 2. Cluster 1 showed minimum turning rate and meander levels based on precise fit of the cluster range, whereas cluster 3 showed low levels of speed, acceleration, and locomotory rate (Fig. 6c). Maximum stop number was specifically fit to cluster 3. These results suggest that two different movement patterns were present in the early phase after infection. Whereas cluster 1 was characterized by “stiff (i.e., minimal turning rate and meander)” movement, cluster 3 was marked by low activity along with maximized stop number (Fig. 6c).

In the lower group, which consisted of clusters 4, 5, and 6 in the late period, behaviors were also differentiated between infection and control groups. Cluster 4 in the left area of the SOM representing infection was characterized by maximum stop duration and low levels of parameters, including speed, acceleration, and locomotory rate (Fig. 6c). In contrast, high activity was observed in cluster 6 representing control in the later period after infection. Cluster 5 appeared to be a mixture, mainly including movement segments of clusters 2 and 6.

Figure 6d further shows examples of movement tracks (10 s) corresponding to each cluster. Clusters 2, 5, and 6 representing the control showed overall broad and round movement within the arena. However, speed was relatively slow in the early phase in cluster 2 and increased as time progressed in clusters 5 and 6 (Fig. 6c). Cluster 6 showed round movement around the arena, whereas cluster 2 tended to show less round movement within the arena (Fig. 6d). Cluster 5 showed a somewhat similar pattern as cluster 6. Clusters 1, 3, and 4 representing an infected state during the course of infection, showed substantially different movement tracks according to different time phases. As stated above, cluster 1 showed continuous, slow, and stiff movement with minimum direction change, manifested as an “arc” shape movement track in the early phase (Fig. 6d). Cluster 3 was markedly different and showed “zigzag” movement with frequent stops as compared with cluster 1 as well as maximum stop number, high meander, and low speed levels in the same early period. Disease development was further identified with cluster 4 in the late period, which showed numerous small round movements with frequent and long stops in a limited area due to maximum stop duration and low levels of speed and acceleration (Fig. 6c). Fish showing these symptoms displayed quick death in most cases. The results indicate that disease development could be identified based on specific symptoms according to the SOM.

In additional file 1, movement parameters are presented as histograms averaged according to the different clusters of the SOM (Fig. 6a). Clusters 1, 3, and 4 representing the infection group in the early and late periods (gray bars) were significantly different from clusters 2, 5, and 6 presenting the control (black bars) for most parameters. As stated above, the parameters regarding linear movement (e.g., speed and locomotory rate) were mainly separated into two groups. Some parameters could be further classified within the groups. Turning rate and meander showed minimum values in cluster 1, whereas stop number was extremely high in cluster 3. Cluster 4 showed significantly high values for stop duration, turning rate, and meander as compared with cluster 1 and was generally in accordance with cluster 3, except for stop number. Not much difference was observed among clusters 2, 5, and 6 representing control.

Association between parameters was further checked by R mode with the SOM (Fig. 6e). Overall, parameters were associated in a similar pattern among clusters, separating behaviors related to stop, linear, and turning movements. However, some differences were observed in the different clusters. In cluster 1 representing early infection, parameter groups were strongly separated with regards to direction change (i.e., meander and turning rate), stop number/duration, and linear movement (speed and locomotory rate) (Fig. 6e). It is also noteworthy that acceleration was further separated from speed and locomotory rate, indicating that an additive increase in speed (acceleration) is not directly related to the speed magnitude. In fact, the three component behaviors (stop, linear movement, and direction changes) were all separately expressed after infection. In cluster 3, substantial changes in relationships were observed between parameters as compared with cluster 1. Speed, locomotory rate, turning rate, and meander generally showed closer association in the upper group, whereas stop duration and stop number formed a separate group at the bottom. It is noteworthy that stop number and stop duration were not close even though they belonged to the same bottom group, which is contrary to their close association in cluster 1. This suggests a somewhat substantial difference between the two symptoms represented by clusters 1 and 3. In cluster 4 representing the late period after infection, somewhat similar patterns to cluster 3 were observed. This indicates that cluster 4 was a consequence of cluster 3 as time progressed. Overall, movement behaviors of zebrafish infected with bacterial pathogens were presented on the SOM, and different states could be identified according to parameter visualization during the different time periods.

## Discussion

Although there have been numerous reports on pathogen infection from the aspects of molecular biology, immunology, histopathology, and epidemiology [57–59], there have not been many investigations addressing infection-behavior relationships due to complexity of the behavioral data. Our study demonstrated that SOM is feasible for elucidating complex disease development after infection.

Considering the principal characteristics of progressive *E. tarda* infection in fish, corresponding behaviors would be expected according to pathological responses due to systemic inflammation, generalized septicemia, and eventual death [60]. The bacterial pathogen *E. tarda* is virulent to Japanese flounder, goldfish and zebrafish [61–63]. Zebrafish carrying disseminated *E. tarda* show septicemia with an uncontrolled inflammatory response and hemolytic-red spots on the skin along with abnormal behavior [64–66]. Disease symptoms caused by *E. tarda* infection are reported to escalate as time progresses during pathogenesis [67, 68]. Our study elucidated progressive abnormal behaviors in response to pathological development as time progressed (Figs. 5 and 6). Zebrafish infected with *E. tarda* CK41 in this study showed segmental necrosis phenomenon and irregular red spots at the caudal-inoculation site (Fig. 2). Especially, abdominal distention and petechial hemorrhaging of the infection-cout were shown to cause symptoms of infection, including “slowing down” characterized by low levels of speed, acceleration, and locomotory rate as shown in clusters 1, 3, and 4 on the SOM (Fig. 6a-c). Furthermore, necrosis in the tail due to expansion of internal organs further contributed to reduction of fish strength.

In addition to the general behavioral response due to infection (i.e., slowing down activity), this study observed different symptoms according to movement parameters and their associations as presented in the SOM (Fig. 6). In the early period, stop-related behavior (stop number) was maximum in cluster 3, whereas stiff movement with minimal turning rate and meander were observed in cluster 1 as stated above (Fig. 6a-c). The stiff movement in cluster 1 could be attributed to infection in the tail of fish (Fig. 2). Further, herniation and hemorrhaging of the tail might have impaired the ability to change movement. On the other hand, the control group immersed in PBS showed no similarity to cluster 1. The majority of the control was represented by clusters 2, 5, and 6 and not the infection group. Cluster 3, which also occupied the early period, showed substantially different movement patterns from cluster 1. In this case, stop number specifically matched to this cluster according to the SOM in Q mode (Fig. 6c). In R mode, parameter associations were also substantially different between clusters 1 and 3. Stop number was markedly different from stop duration in cluster 3. It is also noteworthy that speed was not directly associated with acceleration (Fig. 6e), indicating a sudden change in speed for impulsive propelling of the body.

It is remarkable that symptoms changed as time progressed in cluster 4 and were characterized by stop-related behavior (Fig. 6c). In this case, stop duration was specifically associated with this cluster. As stop number decreased, stop duration was markedly elongated. Considering most test fish died afterwards, long stop duration may indicate loss of general vitality due to long-term infection. Associations between parameters were not significantly different between clusters 3 and 4, which may indicate that cluster 4 was a continuation of cluster 3 in the early period. However, movement patterns of cluster 1 were not associated with clusters 3 and 4. A substantial number of fish (10 individuals) also died directly after showing the symptoms associated with cluster 1 but not clusters 3 and 4. These results indicate that specific physiological networks were differentially activated to cause a large number of stops but not long stop duration. Currently, it is unknown why such differences in behavior are matched to different physiological networks. Further study is warranted in this regard.

Regarding bacterial administration by injection using a syringe such as intraperitoneal injection and intramuscular injection is a general method used in pathological research of fish [67]. However, injection method for introducing bacterial suspensions immediately induces tissue swelling, affecting fish behavior regardless. To eliminate the possibility of artifacts due to injection, bacterial cells were introduced by immersing fish into a fish tank containing a well homogenized bacterial suspension for a given time period. This IM method is very close to the natural infection route [69]. The infection method in this study could not offer the same bacterial dose each individual fish, resulting in slight individual differences in degree of infection and disease progression. Further study is required regarding standardization of doses given to host fishes for observing response behaviors.

The current study has two important advantages. First, recognition of fish was automatized since zebrafish are small (Fig. 2) and it is hard to identify infected fish by visible observation with the naked eye. Using interfacing technique, movement tracks were efficiently recognized in spatial and time domains. Second, computation method such as SOM was found to be feasible for diagnosing disease symptoms. Therefore, subtle changes in behavior of infected fish can be elucidated in a quantitative manner for the objective diagnosis of infection.

Our study further demonstrated that changes in movement parameters could be useful for diagnosing disease development. For instance, stiff movement characterized by minimal direction change along with frequent stops and high meander could be useful for detecting infection in the early period. Further, “stop” related behaviors could be an indicator of the different states of infection, especially long stop duration in the late period of infection. Moreover, the automatic detection system could be applied to screening or marker-assisted selection of specific parameters or for identifying disease emergence for fishery management in the future. It must be noted that our method is not designed to describe the specific pathological mechanism and the cause of any behavioral response remains to be solved. The current study is the first step in this direction, for monitoring infected fish in laboratory conditions. In the next step, the model could be used with a higher degree of complexity. For instance, after completion of laboratory tests, the aquarium could be installed in controlled aquaculture conditions. Subsequently, the effect of some limiting environmental factors such as temperature could be detected. After this step, a mesocosm could be designed, and placed in an optimal position in fish farms. Subsequently, fish behavior could be further examined in more complex field conditions, under the influence of abiotic (e.g., current, temperature) and biotic (e.g., injury, interference by other individuals) factors. Consequently, a step-by-step procedure could be adopted to effectively accommodate high degree of complexity.

## Conclusions

This study provides a basic framework for analyzing the behavior of fish with regard to infectious disease development. Complex response behavior was effectively characterized by SOM in both Q and R modes. Movement of zebrafish was temporally differentiated in early and late infection according to parameter visualization. In early infection, two movement patterns were identified regarding minimal direction change and stop number with higher meander in the forms of stiff movement and numerous small-scale zigzag movements, respectively. Late infection was further characterized by long stop duration. Movement pattern changes would be feasible in diagnosing behavioral disease development. The automatic behavior detection system could be also suitable as a referencing system for diagnosis, screening, and disease management.

## Additional file

Additional file 1:
**Cluster analysis after running SOM.** The vertical bar showed standard deviation. The different alphabets presented significant difference according to the Tukey’s method (*p* <0.05). The numeric number on *x*-axis indicates each cluster in Fig. 6. (PDF 79 kb)
