# Supplementary material for: Analysis of zebrafish (Danio rerio) behavior in response to bacterial infection using a self-organizing map
Source: BMC Vet Res. 2015 Oct 23;11:269. doi: 10.1186/s12917-015-0579-2 (PMC4619490; doi:10.1186/s12917-015-0579-2)

Figure S1. Cluster analysis after running SOM. The vertical bar showed standard deviation. The different alphabets presented significant difference according to the Tukey's method (*p* <0.05). The numeric number on *x* axis indicates each cluster in figure 6.

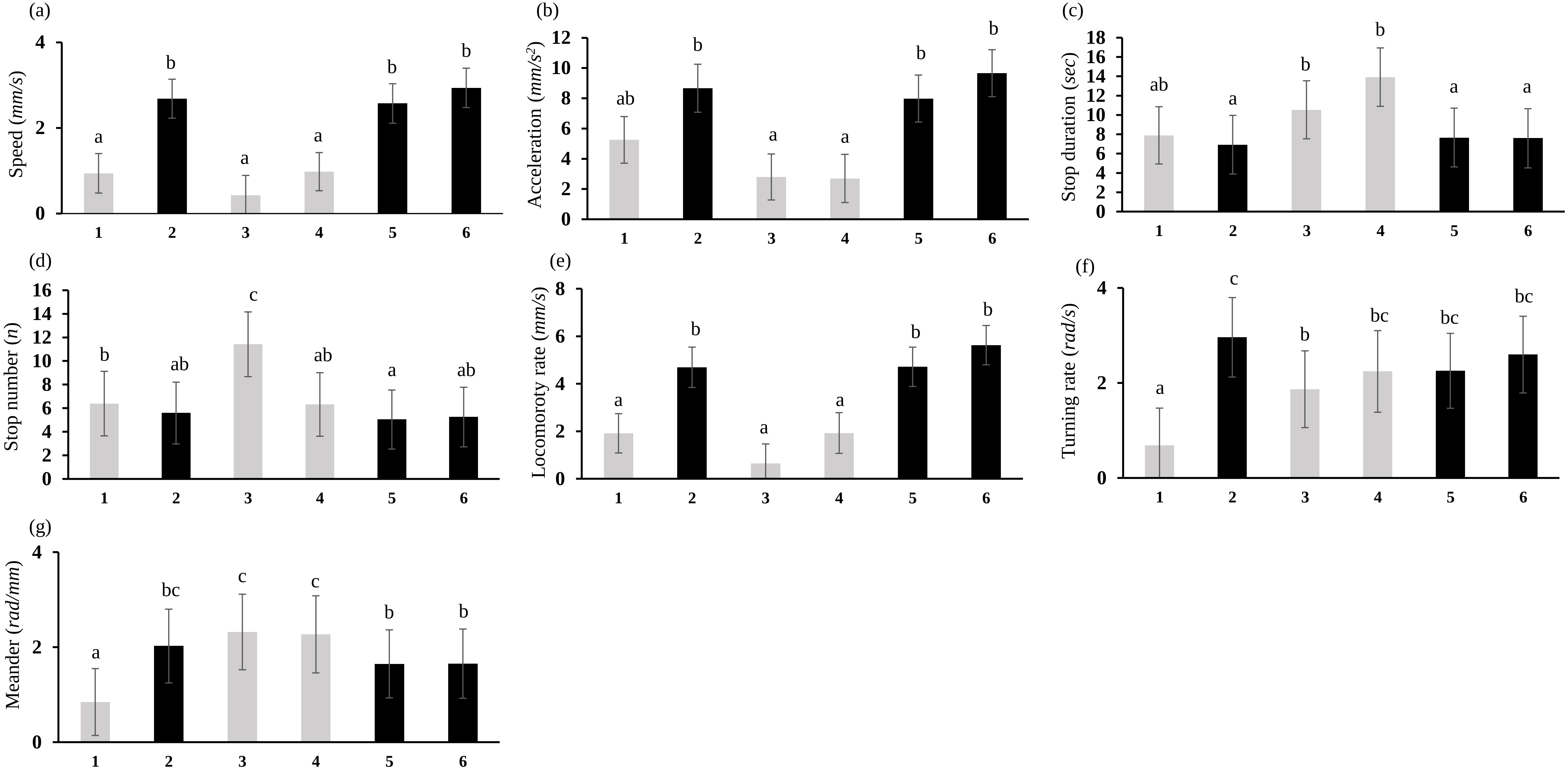

Supplement: Additional file 1: — Cluster analysis after running SOM. The vertical bar showed standard deviation. The different alphabets presented significant difference according to the Tukey’s method (p <0.05). The numeric number on x-axis indicates each cluster in Fig. 6. (PDF 79 kb) [file 12917_2015_579_MOESM1_ESM.pdf]
